# Impact of malaria vector control interventions implemented in Luangwa District southeastern of Zambia: A 13-year observational time series analysis of malaria trends

**DOI:** 10.1371/journal.pone.0336099

**Published:** 2025-11-10

**Authors:** Dingani Chinula, Busiku Hamainza, Japhet Chiwaula, Kenzo Mumba, Reuben Zulu, Ketty Ndhlovu, Samson Kiware, Thomas Reed, Gerry F. Killeen

**Affiliations:** 1 National Malaria Elimination Centre, Chainama Hospital Grounds, Lusaka, Republic of Zambia; 2 School of Biological and Environmental Sciences, University College Cork, Cork, Republic of Ireland; 3 Environmental Health and Ecological Sciences Department, Ifakara Health Institute, Dar es Salaam, United Republic of Tanzania; 4 Sustainability Institute, University College Cork, Cork, Republic of Ireland; Clinton Health Access Initiative, UNITED STATES OF AMERICA

## Abstract

**Introduction:**

Luangwa District has one of the longest running legacy datasets in Zambia regarding reliable monitoring of confirmed malaria cases through the national Health Management Information System (HMIS). It was also one of the first districts to achieve sustained coverage with long-lasting insecticidal nets (LLINs) and indoor residual spraying (IRS) of insecticides.

**Methods:**

HMIS data from2009 to 2021 were analysed using generalized linear mixed models, to assess the effects of LLINs and IRS on rates of inpatient admissions with severe malaria and total confirmed malaria cases. IRS treatments included the pyrethroids deltamethrin and lambda-cyhalothrin, the organophosphate pirimiphos-methyl as emulsifiable concentrate and micro-encapsulated formulations (PM-EC and PM-CS, respectively) and a deltamethrin coformulation with the neonicotinoid clothianidin (DC).

**Results:**

IRS with PM-CS reduced both inpatient admissions (Relative rates (RR) and 95% confidence intervals (CI) for ≤3 months, 4–6 months and 7–12 months post spray = 0.28 [0.19, 0.98] (P = 0.0019), 0.46 [0.31, 0.96] (P = 0.0346) and 0.41 [0.35, 0.94] (P = 0.0174), respectively), and total cases (RR [95%CI] = 0.25 [0.01, 0.67] (P = 0.0017), 0.66 [0.11, 0.88] (P = 0.0087) and 0.48 [0.28, 0.96] (P = 0.0018) for the same post-spray intervals, respectively) for a full year. Furthermore, while reductions of inpatient admissions with severe malaria could only be attributed to DC for the first three months after spraying (RR [95% CI] = 0.27 [0.10, 0.68], P = 0.0379), impacts upon total malaria cases were also apparent for a full year (RR [95% CI] for 1–3 months, 4–6 months and 7–12 months post spray = 0.15 [0.10, 0.68] (P = 0.0017), 0.23 [0.05, 0.56] (P = 0.0013) and 0.43 [0.25, 0.86], P = 0.0029, respectively). Overall, there were >90% fewer inpatient admissions and >80% fewer cases by the end of the study, much of which could be attributed to the immediate effects of scaling up IRS with PM-CS or DC in late 2014 (RR [95% CI = 0.36[0.26,0.51] per year, P = 0.0001) and simultaneously almost doubling the number of health facilities across the district in mid-2016 (RR [95% CI = 0.85 [0.76,0.96] per year, P = 0.0088).

**Conclusions:**

IRS with durable non-pyrethroid insecticide formulations and improved access to diagnosis and treatment were both clearly associated with substantial incremental reductions of malaria incidence. While no epidemiological effect could be attributed to LLINs, this presumably occurred because coverage was already high at the outset and remained so throughout the study.

## Introduction

Between 2000 and 2022, an estimated 2.1 billion malaria cases and 11.7 million deaths were prevented globally. The majority of these averted cases (82%) and deaths (94%) occurred in the WHO African Region [1]. These significant reductions are largely due to the use of long-lasting insecticidal nets (LLINs) and indoor residual spraying (IRS) [1]. In Zambia, IRS and LLINs are the major vector control tools, supplemented with larval source management (LSM) in places where breeding habitats are few, fixed and findable [2]. Three main vectors have been incriminated in the transmission of malaria across Zambia, namely *An. funestus sensu stricto, An. gambiae sensu stricto* and *An. arabiensis* [3–8]. Among the four types of Plasmodium parasites that can cause malaria in humans, *Plasmodium falciparum*, which causes the most severe forms of malaria, is the most predominant in Zambia, accounting for at least 98 percent of all malaria infections in the country [9–12].

Regarding vector control interventions, Zambia has successfully scaled up LLINs since 2005, and they are now distributed through regular mass campaign every three years, to enable universal coverage across all at-risk populations in country [13–17]. These campaigns are complimented by routine continuous distribution through the expanded programme for immunisation (EPI) and antenatal clinics (ANC) [16,17].

For IRS, annual rounds are usually conducted just before the onset of the rainy season, in October or early November. However, budgetary constraints often constrain coverage to only the most malaria-afflicted parts of the country, and delays sometimes arise from slow release of relevant funding. Successive national malaria indicator surveys show that household coverage with IRS rose from a modest 10% in 2006 to 23% by 2010. This growth then continued reaching 29% in 2015 and culminating in a record high of 39% in 2021 reaching slightly over a million households [18–21]. Starting from 2000 up to 2012, Zambia implemented IRS with a range of various insecticide classes, including pyrethroids like deltamethrin (DM) and lambda-cyhalothrin (LC), the carbamate bendiocarb and the organo-chlorine dichlorodiphenyltrichloroethane [3,13,14]. However, from 2013 to date, IRS in Zambia has been implemented only with non-pyrethroid insecticides. These have mostly included the organophosphate pirimiphos-methyl (PM), the neonicotinoid clothianidin and, more recently, a co-formulation of clothianidin with the pyrethroid DC [5,6,22,23].

The effectiveness of LLINs and IRS is threatened by widespread insecticide resistance to pyrethroids, carbamates and organochlorines in Zambia [3,4,7,24]. To address the challenges posed by insecticide resistance, in line with global plan for insecticide resistance management (GPIRM) [25], the Zambia National Malaria Elimination Programme (NMEP) has already developed a national Insecticide Resistance Management and Monitoring Plan. This national plan guides the selection of multiple complementary insecticides for vector control and deploying them as rotations, combinations or mosaics [2], to prevent, mitigate and manage insecticide resistance evolution over time [25,26]. Given the already documented resistance of the main malaria vectors to pyrethroids [3,4,7], Zambia’s current policy is to use complementary insecticides from other classes for IRS when it is used to supplement LLINs [2]. Also, to further mitigate the impact of pyrethroid resistance, in 2020 the NMEP switched from standard pyrethroid-only LLINs to nets that combine pyrethroids with the synergist piperonyl butoxide [17].

Therefore, as Zambia implements various forms and combinations of these key malaria vector control interventions. it is essential to evaluate their impact over time, based on scalable routine epidemiological indicators of incidence. This study therefore makes use of legacy data from the Health Management Information System (HMIS) of the Ministry of Health of the Republic of Zambia. The data is sourced from Luangwa District in the southeast of the country, where this routine surveillance platform has been one of the longest established. It is then used to assess the epidemiological effects of the variable vector control intervention packages implemented upon the incidence of malaria cases confirmed at health facilities across the district over a period of 13 years. This retrospective observational time series analysis specifically examines the impact of LLINs, IRS with several different insecticidal products, and all the various combinations thereof, upon the incidence rates. It focuses on both routine uncomplicated malaria cases diagnosed at nine standard health facilities and more severe cases admitted to the only two hospitals in the district between 2009 and 2021.

## Methods

### Study setting and design

**Fig 1:**
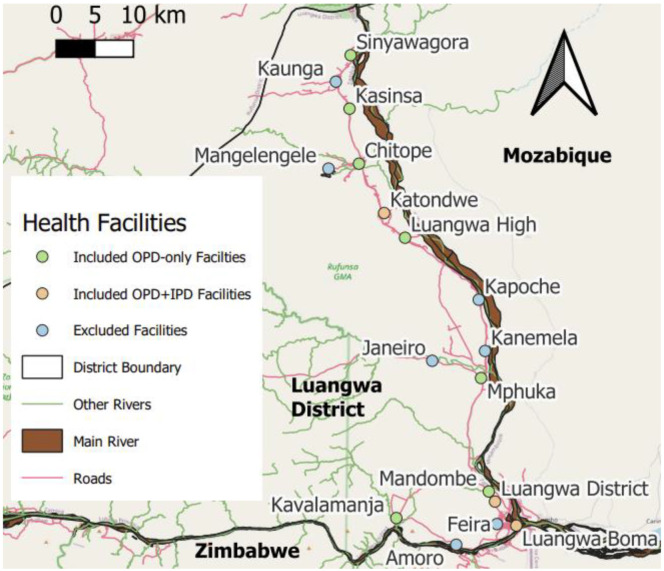
A map of Luangwa District in southeastern Zambia, illustrating the location of all the Health facilities included in the study, as well as those that were excluded because they were only established well after the beginning of the study period (Amoro Health Post, Feira Health Post, Luangwa District Hospital, Janeiro Health Post, Kanemela Health Post, Kapoche Health Post, Mangelengele Health Post and Kaunga Health Post were all opened simultaneously in mid-2016). This map was produced with QGIS® version 3.28.15 open-source software, using information from OpenStreetMap and OpenStreetMap Foundation which is made available under the Open Database License.

This is a retrospective observational evaluation conducted in Luangwa District, southeast of Lusaka in the Republic of Zambia (Fig 1), where malaria transmission is robustly endemic [7,27–30]. The district experiences total annual rainfall ranging from 600 to 800 mm and mean daily maximum temperatures of 35°C, with a single rainy season running from the end of December or early January through to the middle of April. As a result, although malaria transmission is perennial and occurs all year round, it is nevertheless highly seasonal, usually peaking from April to June. *Plasmodium falciparum* is the predominant parasite of humans in the area, the main vector for which has been nominate *An. funestus* *s*. *s*. Although *An. arabiensis* and perhaps even its sibling species *An. quadriannulatus* also contribute to residual transmission [5,7,27–30]. The population of people in the district was approximately 35,000 inhabitants according to the latest 2022 Zambia population census [31], with the most common livelihoods being subsistence farming and fishing.

using a base map obtained from OpenStreetMap® under the Open Database License. OPD: Outpatient department. IPD: Inpatient department.

Luangwa District was an early recipient of LLINs through free distribution campaigns, starting with over 3,200 nets in 2003. This was followed by approximately 2,100 and 7,000 LLINs, respectively distributed free of charge in November 2005 and February 2006, as part of a national campaign to provide one net to every household in the country. In total, over 16,000 LLINs were distributed from 2005 through to 2006, equivalent to approximately three nets per household [32]. Subsequently, from 2008 to 2013, over 32,000 LLINs were distributed through mass campaigns. This was followed by further LLINs mass distribution campaigns that took place in 2014, 2017 and 2020, all of which aimed to deliver one net for every two people to each household. In addition, access to LLINs was further facilitated through routine continuous distribution channels, such as expanded programs for immunization (EPI) and antenatal clinics (ANC) for pregnant women.

The LLINs that were distributed from 2005 to 2020 were mainly PermaNet 2.0® by Vestergaard Frandsen, containing the pyrethroid deltamethrin. Additionally, Olyset® nets from Sumitomo, incorporating the pyrethroid permethrin, were also widely distributed LLIN product during this period. However, from 2020 onwards, procurement policy shifted for both mass campaigns and continuous distribution channels, from ordinary LLINs treated only with pyrethroids to products containing both a pyrethroid insecticide, specifically deltamethrin, and the synergist piperonyl butoxide (PBO), beginning with PermaNet 3.0® by Vestergaard Frandsen. In addition, other LLINs co-treated with the synergist PBO included Olyset® Plus by Sumitomo, which contains permethrin, and Veeralin® by VKA Polymers Pvt Ltd, which contains alpha-cypermethrin were also introduced from 2020 onwards. This change was necessitated to mitigate against the widespread resistance to pyrethroids that was evident throughout the country by that time [3,4,7,33].

In addition, this setting also received IRS with various insecticide products, which was initially externally funded and implemented by private sector contractors engaged by those funders (United States Agency for International Development and Global Fund to Fight AIDS, Tuberculosis and Malaria) from 2010 through to 2012. From 2013 onwards, IRS was fully funded and implemented by the Government of the Republic of Zambia thereafter. In 2010 and 2011, IRS was implemented using pyrethroids, specifically a wettable granule (WG) formulation of DM (DM-WG; K-Othrine WG® 250, Bayer Environmental Science) and a capsule suspension (CS) formulation of LC (LC-CS; Icon 10CS®, Syngenta). Although IRS was almost always applied at or just before the onset of the rainy season, in October or early November, the round planned for 2012 was implemented several months late. It was eventually carried out in February 2013, using the short-lived emulsifiable concentrate (EC) formulation of PM (PM-EC; Actellic 50EC®, Syngenta) [34,35]. The switch to this organophosphate was necessitated by detection of physiological resistance to pyrethroids in this area [3,7]. In later years, starting from 2014 up to 2019, IRS was implemented with the much longer lasting capsule suspension (CS) formulation of PM (PM-CS; Actellic 300CS®, Syngenta) [35–37]. From 2020 and 2021, there was then a shift from PM-CS to a long-acting insecticide combination formulation containing both deltamethrin and clothianidin (DC; Fludora Fusion®, Envu).

### Data sources for epidemiological outcomes

The NMEP relies heavily on a robust malaria surveillance platform to monitor the impact of malaria interventions that have been implemented over time. The NMEP collates routine malaria data generated by health facilities through the Health Management Information System (HMIS) database. This database is used to continuously monitor indicators of service delivery and disease burden that inform programming and policy on an ongoing basis while the Malaria Rapid Reporting System (MRRS) complements HMIS by capturing data generated at community level through Community Health Workers (CHWs). However, the MMRS platform has not yet been rolled out in Luangwa, despite it being one of the first districts where it was first piloted [7,38].

The programmatic legacy data included in this retrospective observational study therefore all come from the HMIS platform, running from 2009 through to 2021. Unfortunately, data available from before 2009 had to be excluded because so much data were inappropriately reported or entirely missing: Before 2009, most facilities did not reliably report to HMIS, and the few that did only did so on a quarterly basis [39]. Post 2021 data were excluded from the analysis because an operational pilot of micro-mosaic application of IRS was ongoing in Luangwa over that period, making it difficult to compare with what happened previously.

All disease burden data were extracted from HMIS, with the malaria incidence outcomes considered to be the monthly totals of malaria cases among patients of all ages. These cases were confirmed with either rapid diagnostic tests or microscopy, as outpatients at any of the nine facilities that operated over the full course of the study period.

Inpatient cases were recorded as those admitted to either one of the only three health facilities that had the capacity to provide inpatient care in the district (Fig 1), namely (1) Katondwe Mission Hospital, (2) Luangwa Boma Rural Health Centre and (3) the new Luangwa District Hospital that was opened in July 2016. In order to avoid disruption to time trends in these inpatient data by the opening of the new district hospital and the resulting redistribution of these severe cases across these two facilities in close proximity to each other, total cases from both facilities for each month were pooled from July 2016 onwards, with the pooled total from the two (or from the single facility that preceded them) reported under the single name *Luangwa District Hospital* for simplicity and clarity.

At present, Luangwa District now has a total of 17 health facilities, which includes two hospitals, nine health centres and six rural health posts (Fig 1). Notably, however, eight of these are relatively new, having all been simultaneously opened in mid 2016. Correspondingly, no long-term trends in malaria incidence at these facilities could be assessed, so the outpatient data reported by them were excluded from consideration in this analysis (Fig 1).

### Data sources for intervention delivery and environmental variables

The vector control data was obtained from programmatic sources (NMEP), dating back from as far back as 2005. To account for climatic variables, rainfall and temperature data were obtained from the Zambia Metrological Department, starting from 2005 up to 2021.

### Data analysis

**Fig 2:**
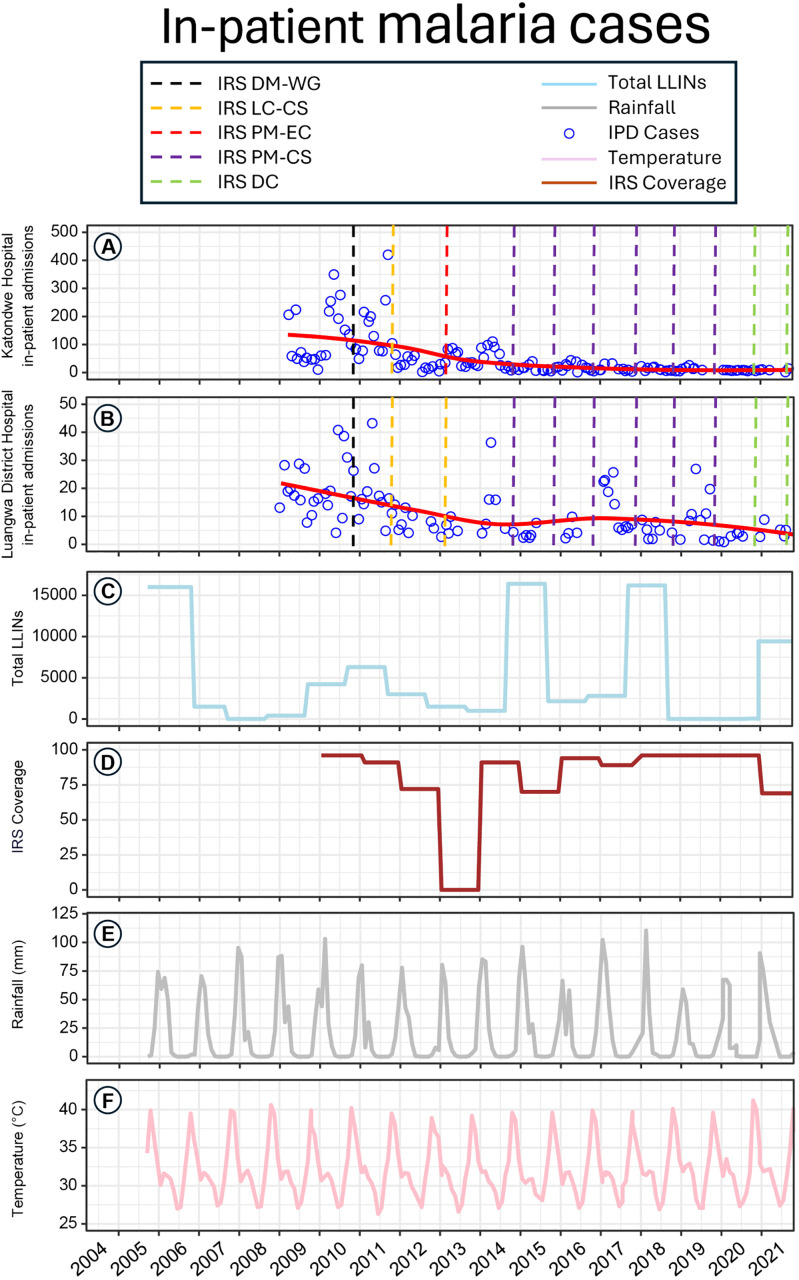
Time series plots of malaria inpatient admissions (A) Katondwe Mission Hospital and (B) Luangwa District Hospital, as well as recorded district level (C) total LLINs delivered that year (D), IRS coverage rates for that year, (E) monthly total rainfall and (F) monthly mean daily maximum temperature, with the predominant IRS treatments in their catchment areas indicated by coloured vertical bars.

**Fig 3:**
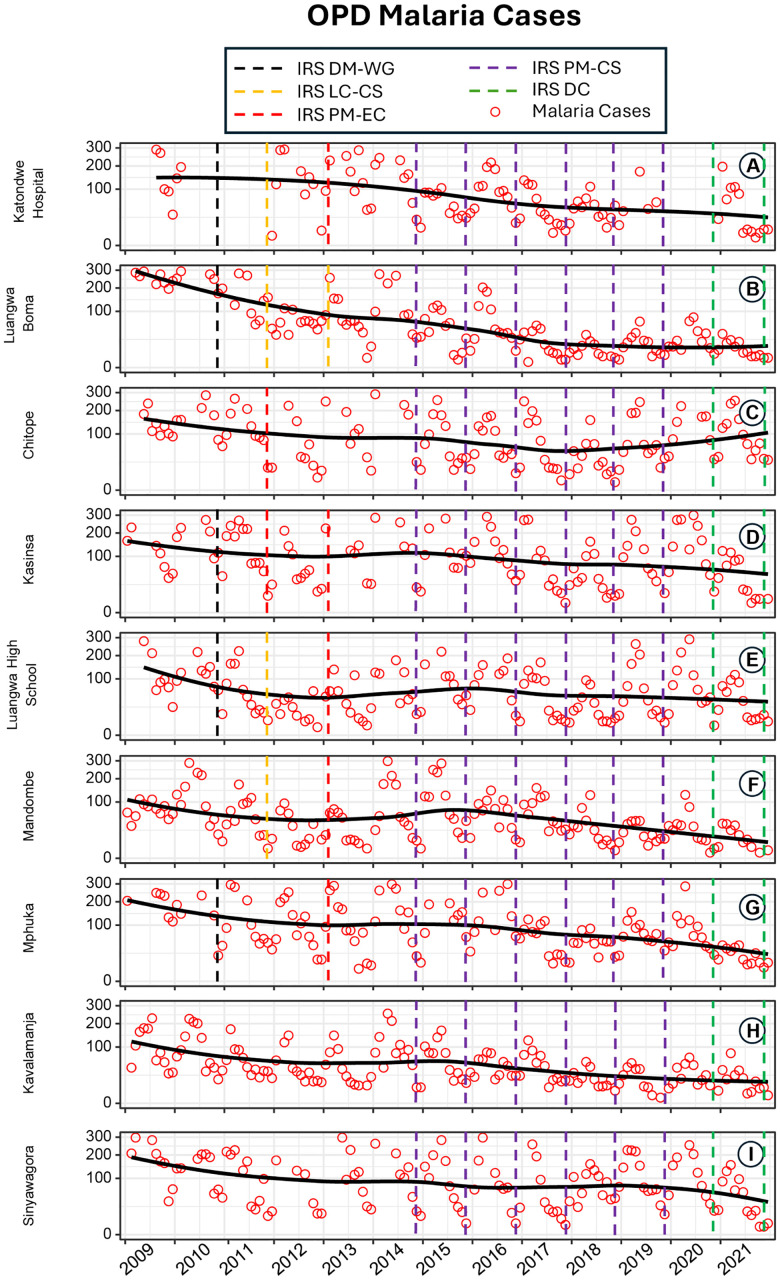
Time series plots of all confirmed outpatient malaria cases recorded at facilities with catchments sprayed with various IRS treatments, namely (A) Katondwe Mission Hospital, (B) Luangwa Boma Rural Health Centre, (C) Chitope Rural Health Centre, (D) Kasinsa Rural Health Centre, (E) Luangwa High School Rural Health Centre, (F) Mandombe Rural Health Centre, (G) Mphuka Rural Health Centre, (H) Kavalamanja Rural Health Centre and (I) Sinyawagora Rural Health Centre, with the predominant IRS treatments in their respective catchment areas indicated by coloured vertical bars.

The map presented in Fig 1 was generated using QGIS® version 3.28.15, an open-source geographic information system, incorporating a base layer sourced from OpenStreetMap® and licensed under the Open Database License. All analyses were performed with R version 4.3.3 open-source software, using the *RStudio (2023.12.1 + 402)* environment*.* Exploratory descriptive analyses of the raw data for each health facility were carried out using the *ggplot2* package to check for obvious anomalies and identify broad trends. In addition, time series plots of malaria cases alongside climatic variables were plotted to confirm and examine expected seasonal cycles in malaria incidence driven by rainfall and temperature (Fig 2 and 3). It was apparent from these visual graphical assessments that these raw data were over-dispersed, and this was confirmed by noting that their variances were greater than their means.

To estimate the number of LLINs distributed per person per year, total numbers of nets delivered to the district in the previous 12 months were divided by the estimated total district population for that year. However, LLINs are expected, in principle, to last for at least three years [40], so the regression analyses described below needed to also account for potential effects of nets distributed over several previous years. This variable (Total LLINs distribution ratio per person in the last 12 months) was therefore also recoded into three derived variables representing a series of three temporal lags staggered by one year: Over annual cycle one year ago, over annual cycle two years ago and over annual cycle three years ago.

For each of the various IRS treatments applied at various times (DM-WG, LC-CS, PM-EC, PM-CS and DC), the interventions were categorised based on the specific product used. Each treatment was then coded into three sequentially lagged post-application time periods: 1–3 months, 4–6 months and 7–12 months since beginning of the last spray round in which that particular IRS treatment was applied. This approach to allowing for the decay of IRS efficacy over time was not only based on evidence from a range of entomological evaluations of such products [8,41]. But has also proven effective in previous epidemiological assessments of IRS effectiveness in this same setting [7].

To assess the effects of environmental and intervention-related independent variables upon malaria incidence, generalised linear mixed models (GLMM) with an intercept were fitted in R with the *glmmTMB* package. A type II negative binomial distribution was specified for this dependent variable, because this proved to be superior to Poisson or type I negative binomial distributions in terms the Akaike Information Criterion (AIC) for goodness of fit. To account for covariance arising from repeated measures made at individual health facilities with fixed catchment areas, and from naturally occurring dependence of malaria incidence at any given facility upon rates in the same place in the recent past. Health facility was treated as a random effect, within which the number of months since the start of the study period was nested to allow for first order temporal autocorrelation over such short-term timescales.

Best-fit multiple regression models were then identified through a two stage, forward stepwise selection procedure that first optimized these GLMMs based on the most important environmental variables. The effects of vector control interventions were then incorporated using a similarly objective and systematic set of rules for sequential inclusion/exclusion of candidate explanatory variables.

Prior to accounting for the effects of LLINs and IRS, models included only climatic variables, specifically temperature, monthly mean rainfall, and various plausible monthly lags thereof, were evaluated and optimized. First, each and every potentially predictive variable for either inpatient or outpatient malaria incidence was assessed by including it by itself in a simple single predictor variable analysis. More complex models were then developed, by adding additional predictors in order of the goodness of fit estimates obtained for each in the single predictor analysis. Predictors that did not prove significant in the multiple-predictor model or improve its goodness of fit were subsequently removed. The order in which independent variables were added to the multiple predictor models was determined based on goodness of fit, with the lowest AIC values added first.

Once this parsimonious best fit model of environmental effects was identified, the various variables potentially capturing vector control intervention effects were added one at a time. This yielded a series of two predictor (incidence of inpatient admissions) or three predictor (incidence of outpatient cases) models, each giving a preliminary indication of the predictive value of each of these potential independent variables. For this second stage of forward stepwise selection, all these intervention-related variables were added one at a time to the multiple predictor model, in reverse order of the AIC estimates for their respective simpler models. As in the first stage, variables were then removed if they neither proved significant nor improved goodness of fit, exactly as described for the first stage.

However, an overall downward trend was observed in graphs of malaria incidence rates over the long term that appeared to precede the introduction of IRS in almost all of the nine different catchments monitored. Also, after a brief period of stagnation in the middle of the study, a second steady downward trend is apparent over the latter five or six years. An interrupted time series analysis of outpatient cases was therefore undertaken, to assess how well these public health gains could be attributed to the immediate or longer-term effects of introducing district-wide IRS with durable non-pyrethroid insecticide formulations (PM-CS or DC) in late 2014, the immediate or longer-term effects of simultaneously opening so many new health facilities in mid-2016, or any overall long-term trends unrelated to either IRS or the opening of so many new health facilities. To conduct this complementary analysis, the rainfall and temperature terms in the multiple-predictor regression model described immediately above were retained but all the IRS intervention terms were simplified to a single binary form, specifically whether any non-pyrethroid IRS product that was identified as providing clear protection over one full year in the preceding analysis described above (PM-CS and DC) had been deployed in the previous 12 months. Furthermore, two empirical time trend variables were introduced, specifically decimal forms of the number of years since either the study started or since these more effective IRS treatments were first introduced across the district. Also, similar terms for the immediate and longer-term effects of simultaneously opening the 8 new health facilities were considered as independent variables with potential to influence the overall trajectory of malaria incidence. Similarly to all the analyses described above, the most parsimonious form of that multiple-predictor regression model was also then identified through forward stepwise selection. Note, however, that the best fit model identified based on purely statistical criteria, which also included the immediate effect of IRS with PC-CS or DC, indicated an implausible steady increase of incidence after IRS with PC-CS or DC was introduced and an exaggerated subsequent effect of opening the new health facilities over time (See *Results*). This best fit model with three independent variables of interest was therefore considered to be most probably spurious in nature, with the fitted log-linear effects of two temporal variables that become relevant at different time points less than two years apart coincidentally explaining a nuanced, uncertain time trend with a non-linear trajectory than either variable alone. The final model reported therefore reverts to the best of the models with only two explanatory variables of interest, namely the immediate effects of district wide scale up of IRS with PM-CS and then DC from late 2014 onwards and the longitudinal effect over time of simultaneously opening so many new facilities across the district in mid-2016.

**Ethical considerations:** This study did not use any identifiable individual-level patient data, but rather retrospective HMIS data aggregated and reported at health facility level. As such these are not collected for research purposes *per se* but rather for routine impact evaluations of malaria control interventions that are regularly conducted and disseminated by the Zambia National Malaria Elimination Programme.

## Results

### Inpatient admissions with severe malaria

In 2009, total inpatient admissions at Katondwe Hospital totaled over eight hundred severe malaria cases, and that more than doubled in 2010 and in 2011 (Fig 2A). This baseline incidence rate for severe malaria is remarkably high, equivalent to between 3% and 6% of the entire district’s population being admitted to this one hospital as inpatients each year. However, this very high baseline rate of severe malaria then steadily declined from 2012 through to 2021, at which point total annual inpatient admissions had remained consistently below 100 cases for three years (Fig 2A). A similar long-term picture of a generally steady long-term decline from 2012 onwards was observed for Luangwa District Hospital. However, it began with much lower baseline incidence rates for severe malaria and experienced some notable seasonal spikes in 2014, 2017 and 2019 (Fig 2B). Overall, malaria annual inpatient admissions for the entire district declined to less than 100 by 2021, representing an overall reduction of at least 90% compared to the starting point of over 1000 in 2009.

Although this long-term decline appeared to begin within two years of the introduction of IRS, the seasonal peak of incidence in early 2011 was similar in scale to that for the previous year and double that for 2009, despite following soon after that first spray round in late 2010. Furthermore, a series of LLINs distributions between 2008 and 2013 that peaked in 2010 also coincided with the onset of this long-term decline, so it is not obvious from Figs 2A, B, C and D that this downward trend was associated with either of these interventions in particular. While the long-term decline at Katondwe Hospital appeared consistently steady, all three apparent resurgences of severe malaria at Luangwa District Hospital were associated with temporal gaps in vector control intervention delivery: a lack of IRS in 2014, a two-year lag since the last mass ITN distribution in 2017 and a one-year lag in 2019. Similarly to the overall long-term trend, these occasional short-term resurgences could not, consequently, be descriptively attributed to coverage gaps for either intervention.

A GLMM analysis was therefore conducted to determine whether epidemiological effects could be statistically attributed to either of these vector control interventions or both. First and foremost, however, these regression models were used to account for the effects of highly dynamic environmental variables, specifically rainfall and temperature (Figs 2E and F, respectively). While single predictor analysis indicated that almost all these variables were associated with inpatient malaria incidence rates, monthly total rainfall two and three months previously was included as an independent variable in the final best-fit multiple predictors model (Table 1).

**Table 1:** Single-predictor and multiple-predictor regression analysis of the dependence of the monthly total numbers of confirmed malaria-related inpatient admissions at Katondwe Mission Hospital and Luangwa District Hospital (Fig 1) upon environmental variables and vector control interventions. For details of how these generalized linear mixed models (GLMMs) were fitted to these rates of severe malaria incidence and then optimized through a two-stage forward stepwise selection procedure, see the *Methods* section. Terms significant at the 5% level are highlighted in bold.

Independent variables (predictor)	Single predictor		Multiple predictors	
	**RR [95% CI]**	**P**	**RR [95% CI]**	**P**
*Environmental Variables*				
Total Rainfall this month(mm)^*b*^	**1.00 [0.99,1.00]**	**0.0348**	1.00 [0.99,1.00]	0.0736
Total Rainfall 1 month ago(mm)^*b*^	**1.01 [1.00,1.01]**	**<0.0382**	1.00 [1.00,1.01]	0.2368
Total Rainfall 2 months ago(mm) ^*a*^	**1.01 [1.00,1.01]**	**<0.0001**	**1.00 [1.00,1.01]**	**0.0267**
Total Rainfall 3 months ago(mm)^*a*^	**1.01 [1.00,1.01]**	**<0.0001**	**1.00 [1.00,1.01]**	**0.0211**
Total Rainfall 4 months ago(mm) ^*a*^	**1.01 [1.00,1.01]**	**<0.0001**	1.00 [1.00,1.01]	0.0881
Temperature(°C)^*b*^	**0.97 [0.93,1.00]**	**0.0362**	0.98 [0.95,1.01]	0.1912
*Total district LLIN distributions* ^*b*^				
Over preceding annual cycle	0.41 [0.12,1.38]	0.1508	0.85 [0.21,3.43]	0.8188
Over annual cycle 1 year ago	0.85 [0.16,4.68]	0.8544	2.17[0.36,12.99]	0.3964
Over annual cycle 2 years ago	0.91 [0.18,4.47]	0.9073	1.92[0.35,10.68]	0.4563
Over annual cycle 3 years ago	1.30 [0.43,3.95]	0.6398	1.42 [0.45,4.44]	0.5479
*IRS-Deltamethrin wettable granule (DM-WGP)* ^*a*^				
≤3 months since last spray	0.79 [0.42,1.50]	0.4765	0.80 [0.44,1.47]	0.4714
4 to 6 months since last spray	1.23 [0.61,2.49]	0.5604	1.24 [0.64,2.40]	0.5326
7 to 12 months since last spray	**1.93 [1.08,3.44]**	**0.02678**	**1.94 [1.13,3.34]**	**0.0173**
*Pirimiphos-methyl capsule suspension (PM-CS)* ^*a*^				
≤3 months since last spray	**0.38 [0.20,0.74]**	**0.0044**	**0.28 [0.14,0.57]**	**0.0019**
4 to 6 months since last spray	0.64 [0.34,1.23]	0.1808	**0.46 [0.22,0.94]**	**0.0346**
7 to 12 months since last spray	0.58 [0.31,1.11]	0.0993	**0.41 [0.20,0.85]**	**0.0174**
*IRS-Pirimiphos-methyl emulsifiable concentrate (PM-EC)* ^b^				
≤3 months since last spray	0.86 [0.45,1.64]	0.6503	1.16 [0.60,2.24]	0.7136
4 to 6 months since last spray	0.71 [0.35,1.45]	0.3487	0.87 [0.44,1.74]	0.5766
7 to 12 months since last spray	0.57 [0.28,1.15]	0.1169	0.64 [0.33,1.26]	0.1473
*IRS-Deltamethrin plus clothianidin (DC)* ^*a*^				
≤3 months since last spray	0.61 [0.18,2.05]	0.4277	**0.27 [0.08,0.93]**	**0.0379**
4 to 6 months since last spray	1.51 [0.48,4.76]	0.4814	0.62 [0.18,2.09]	0.4384
7 to 12 months since last spray	0.90 [0.29,2.79]	0.8493	0.38 [0.12,1.23]	0.1069
*Lambda-cyhalothrin capsule suspension (LC-CS)* ^*b*^				
≤3 months since last spray	0.84 [0.39,1.80]	0.6595	1.03 [0.48,2.22]	0.9353
4 to 6 months since last spray	0.67 [0.26,1.71]	0.4019	0.78 [0.31,1.93]	0.6237
7 to 12 months since last spray	0.55 [0.19,1.61]	0.2782	0.60 [0.21,1.68]	0.3655

RR: Relative Rate ratio (calculated by taking the exponent of the corresponding coefficient from the GLMM, which used a log-link function).

CI: Confidence interval.

^a^Parameters included in the final model.

^b^Parameters not included in the final model

Regarding interventions, neither annual rates of LLIN delivery over the preceding year, nor any yearly time lag thereof before that, were included in the final multivariate model for inpatient admissions (Table 1). This indicates that no epidemiological benefit could be statistically attributed to fluctuations in LLINs supply over the course of the study.

For IRS, the final best fit multi-predictor model indicates PM-CS apparently reduced malaria inpatient admissions for an entire year after application. This long-lasting organophosphate product apparently reduced severe malaria by nearly three quarters in the first 3 months and by more than a half for 4–12 months after spraying (Table 1). While IRS with DC was associated with apparent reductions of severe malaria by almost three quarters over the first three months after spraying, no effect beyond that brief post-application period could be detected. In contrast, neither PM-EC nor LC-CS had any apparent effect on inpatient admissions at any time after spraying. Surprisingly, IRS with Deltamethrin WG was apparently associated with a doubling of inpatient admissions from 7 to 12 months after application. While the most obvious interpretation is that this may well be a spurious model fit, it is notable that similarly counterintuitive trends have been obtained for mosquito densities and diagnostic positivity rates reported by community health workers in previous studies [7].

### Routine cases of uncomplicated malaria at outpatient facilities

For the incidence of routine malaria cases handled as outpatients, there was considerable variation in intervention regime and year-on-year time trends across the nine separate facilities (Fig 3). Consistent with programmatic rationale at the time, IRS appears to have first reached the catchments of facilities with the highest incidence rates between 2010 and 2013, with blanket coverage of the whole district only being achieved from 2014 onwards.

Overall, the district as a whole progressed from a reported total incidence of confirmed outpatient malaria cases of almost 26,000 in 2009, to slightly over 14,000 by 2013. This was followed by a slight spike in 2014 after a gap in IRS coverage, and then a somewhat bumpy general decline down to only 4,422 cases across all nine health facilities in 2021. This translates into an overall long-term reduction of over 80% (Fig 3).

However, because outpatient malaria cases across the district seemed to exhibit a general downward trend even before IRS deployment (Fig 3), it was not possible to descriptively attribute this impressive long-term decline to the IRS alone. While a gentle downward trajectory was apparent in most catchments prior to IRS introduction (Fig 3), it was particularly clear in catchments that were only reached when the government supported scale up of blanket coverage with PM-CS across the whole district in 2014. It is therefore unclear from Fig 3 how much IRS contributed to the observed overall alleviation of malaria burden over the long term. Having said that, it is notable that an apparent resurgence of malaria incidence occurred in 2014 in several of the catchments that had been reached earliest with IRS but then never received it that particular year. This subtle rebound effect appears to descriptively confirm the importance of IRS in sustaining malaria case reductions when implemented as a supplementary intervention to LLINs.

Multi-predictor regression analysis revealed that variations in both temperature and rainfall contributed to the erratic nature of month-by-month trends in uncomplicated malaria incidence. The effect of rainfall was particularly strong, with incidence closely associated with precipitation one, two, three and four months previously (Table 2). However, although malaria incidence was highly seasonal as a result, annual means and peaks remained quite consistent at each health facility until after IRS was introduced (Fig 3).

**Table 2:** Single-predictor and multiple-predictor regression analysis of the dependence of the monthly total numbers of confirmed malaria cases handled as outpatients at all nine facilities (Fig 1) upon environmental variables and vector control interventions. For details of how these generalized linear mixed models (GLMMs) optimized through a two-stage forward stepwise selection procedure, see the Methods section. Terms significant at the 5% level are highlighted in bold.

Independent variables (predictor)	Single predictor		Multiple predictors	
	**RR [95% CI]**	**P**	**RR [95% CI]**	**P**
*Environmental Variables*				
Total Rainfall this month (mm) ^*b*^	1.00[1.00,1.00]	0.3160	1.00 [1.00,1.00]	0.3633
Total Rainfall 1 month ago (mm) ^*b*^	**1.00[1.00,1.01]**	**<0.0001**	**1.01 [1.00,1.01]**	**<0.0001**
Total Rainfall 2 months ago(mm) ^*a*^	**1.01[1.00,1.01]**	**<0.0001**	**1.00 [1.00,1.00]**	**<0.0001**
Total Rainfall 3 months ago(mm) ^*a*^	**1.00[1.00,1.01]**	**<0.0001**	**1.01 [1.00,1.01]**	**<0.0001**
Total Rainfall 4 months ago(mm) ^*a*^	**1.00[1.00,1.01]**	**<0.0001**	**1.01 [1.00,1.01]**	**<0.0001**
Temperature (°C)^*a*^	**0.94[0.89,0.99]**	**<0.0001**	**0.97 [0.97,0.99]**	**<0.0001**
*Total district LLIN distributions* ^*b*^				
Over preceding annual cycle	0.62[0.30,1.27]	0.1900	1.19 [0.59,2.40]	0.6278
Over annual cycle 1 year ago	0.38[0.13,1.07]	0.0669	1.13 [0.41,3.11]	0.8134
Over annual cycle 2 years ago	0.50[0.18,1.36]	0.1735	1.28 [0.49,3.38]	0.6175
Over annual cycle 3 years ago	0.65[0.33,1.30]	0.2260	1.00 [0.53,1.90]	0.9934
*IRS-Deltamethrin wettable granule (DM-WG*^*b*^)				
≤3 months since last spray	0.85[0.48,1.52]	0.5899	0.63 [0.40,1.01]	0.0566
4 to 6 months since last spray	1.28[0.67,2.44]	0.4501	0.97 [0.53,1.78]	0.9322
7 to 12 months since last spray	1.36[0.78,2.38]	0.2777	1.05 [0.58,1.90]	0.8611
*Pirimiphos-methyl capsule suspension (PM-CS)* ^*a*^				
≤3 months since last spray	**0.52[0.38,0.70]**	**<0.0001**	**0.25 [0.18,0.34]**	**<0.0001**
4 to 6 months since last spray	1.35[0.98,1.85]	0.0682	**0.66 [0.48,0.89]**	**<0.0001**
7 to 12 months since last spray	1.02[0.76,1.38]	0.8744	**0.48 [0.35,0.65]**	**<0.0001**
*IRS-Pirimiphos-methyl emulsifiable concentrate (PM-EC)* ^*b*^				
≤3 months since last spray	0.63[0.33,1.20]	0.1640	0.63 [0.36,1.12]	0.3052
4 to 6 months since last spray	1.08[0.58,2.03]	0.8070	0.78 [0.44,1.39]	0.4045
7 to 12 months since last spray	0.73[0.39,1.37]	0.3310	0.77 [0.47,1.27]	0.1142
*IRS-Deltamethrin plus clothianidin (DC)* ^*a*^				
≤3 months since last spray	**0.29[0.19,0.44]**	**<0.0001**	**0.15 [0.10,0.23]**	**<0.0001**
4 to 6 months since last spray	0.75[0.44,1.29]	0.2980	**0.42 [0.25,0.73]**	**0.0017**
7 to 12 months since last spray	**0.43[0.25,0.74]**	**0.0020**	**0.23 [0.13,0.39]**	**<0.0001**
*Lambda-cyhalothrin capsule suspension (LC-CS)* ^*a*^				
≤3 months since last spray	1.54[0.61,3.87]	0.3640	1.01 [0.41,2.50]	0.9799
4 to 6 months since last spray	1.39[0.50,3.85]	0.5224	1.13 [0.45,2.85]	0.7961
7 to 12 months since last spray	0.48[0.22,1.01]	0.0536	**0.45 [0.23,0.90]**	**0.0230**

RR: Relative rate, calculated by taking the exponent of the corresponding coefficient from the GLMM, which used a log-link function.

CI: Confidence interval.

^a^Parameters included in the final model

^b^Parameters not included in the final model

This regression analysis of the short-term effects of meteorological conditions and vector control interventions upon total case incidence rates also confirmed that IRS implementation, with non-pyrethroids in particular, clearly suppressed malaria burden (Table 2). Similarly to inpatient admissions with severe malaria (Table 1), distributions of LLINs had no statistically attributable influence upon the incidence of routine malaria cases managed as outpatients (Table 2). Again, IRS with the long-lasting organophosphate product PM-CS consistently reduced the outpatient cases for a full 12 months, by three quarters in the first 3 months and by at least a third from 4 to 12 months after spraying (Table 2). IRS with the DC product also greatly suppressed malaria transmission, but these statistically attributable effects persisted for much longer (Table 2) than was apparent for inpatient admissions (Table 1). This is probably because of the greater statistical power attainable: The total of 7,929 severe cases admitted to the two hospitals is 23-fold fewer than the 183,087 routine outpatient cases that occurred across all nine facilities over the full course of the study. The incidence of such routine cases was reduced by approximately 85% over the first 3 months, by over three quarters from 4 to 6 months and by more than half from 7 to 12 months after spraying (Table 2). IRS with the DM-WG appeared to reduce outpatient malaria cases by two fifths over the first 3 months after spraying, although that apparent effect only approached statistical significance and no similar effect was apparent at any time thereafter (Table 2). For the other pyrethroid-based LC-CS product, the only detectable effect was a reduction by over half that was surprisingly only observed for 7–12 months after spraying, which may well simply represent a spurious model fit (Table 2). On the other hand, for the short-lived organophosphate formulation PM-EC, no effect upon malaria case incidence was obvious at any time after spraying (Table 2).

The complementary interrupted time series analysis that followed confirmed that the introduction of IRS with durable non-pyrethroid insecticide formulations, specifically PM-CS at the end of 2014 and then DC from late 2020 onwards) was clearly associated with an immediate 65% reduction of malaria case incidence, underscoring its critical role in suppression of transmission (Table 3). Although exploratory analysis of the subset of data up to late 2014 seemed to confirm the visually obvious downward trend in the data from the outset of the study (P = 0.046), no such effect was included in the final best fit model when the whole dataset was used (Table 3). However, the final best fit interrupted time series analysis model does indicate that a clear downward trend occurred from the middle of the study onwards, and that this steady long-term decrease was most closely associated with the simultaneous opening of 8 new health facilities in mid-2016, rather than the introduction of effective, long-lasting IRS in late 2014 (Table 3). Having said that, it is well known that IRS can take two to three years to achieve its full effects on malaria burden [42–45], so this empirical statistical association could well arise from the coincidence of such delayed impacts with the opening of these new facilities less than two years later. Indeed, a clearly spurious model that included significant effects for both time since these effective IRS treatments were introduced across all HFCAs (Implausible upward trend exceeding 60% per year) and time since the facilities were opened (Implausibly steep downward slope exceeding 50% per year) was disregarded on the basis of implausibility (See *Methods*), indicating considerable uncertainty about the shape and underlying cause of that downward trajectory.

**Table 3:** Single-predictor and multiple-predictor regression interrupted time series analysis of IRS on monthly total numbers of confirmed malaria cases handled as outpatients at all nine facilities. For details of how these generalized linear mixed models (GLMMs) optimized through a two-stage forward stepwise selection procedure, see the *Methods* section (*Methods* section). Note that essentially identical temperature and rainfall terms to those in Table 2 were also included but are not presented here in the interests of brevity and clarity. Terms significant at the 5% level are highlighted in bold.

Independent variable (predictor)	Single predictor		Multiple predictors	
	**RR [95% CI]**	**P**	**RR [95% CI]**	**P**
Years since start of study^a^	**0.85[0.82, 0.89]**	**<0.0001**	0.95[0.90,1.02]	0.1630
IRS with PM-CS or DC introduced^b^	**0.29 [0.22, 0.39]**	**<0.0001**	**0.35[0.24, 0.53]**	**<0.0001**
Years since IRS with PM-CS or DC introduced^a^	**0.78 [0.72, 0.84]**	**<0.0001**	**0.90[0.83,1.00]**	**0.0482**
New facilities opened^a^	**0.54 [0.39, 0.75]**	**0.0002**	0.92[0.66,1.27]	0.6080
Years since new facilities opened^b^	**0.72[0.65, 0.80]**	**<0.0001**	**0.85[0.76,0.96]**	**0.0088**

RR: Relative rate, calculated by taking the exponent of the corresponding coefficient from the GLMM, which used a log-link function.

CI: Confidence interval.

^a^Parameters not included in the final model

^b^Parameters included in the final model

## Discussion

This retrospective analysis of HMIS data evaluated the impact of LLINs and IRS on malaria burden in Luangwa District over 13 consecutive years, allowing for the confounding erratic seasonal influences of temperature and rainfall. Given that the main malaria vector in this area is the slow-growing species *An. funestus.s.s.*, it is perhaps unsurprising that surges of clinical cases were most readily attributed to rainfall one to four months previously, similarly to a number of other settings across in sub–Saharan Africa [46–53]. While high temperatures were observed not to have any effects on inpatient admissions, they were associated with lower numbers of outpatient malaria cases. This counterintuitive association between reduced malaria burden and warmer conditions that accelerate parasite sporogony is, however, not unprecedented [54,55]. Furthermore, it may be readily explained in this case by the fact that the hottest times of the year in this setting are usually also the driest (Figs 2E and F), resulting in fewer breeding habitats being available for mosquito proliferation.

Regarding vector control interventions, this district received a consistent supply of LLINs through mass campaigns and also other continuous distribution channels, such as EPI and ANC over several years [7,32]. Although the statistical analysis presented herein could not attribute any effect upon either inpatient or outpatient malaria cases to LLINs, this finding should be interpreted with caution because large numbers of nets had already been distributed long before these routine epidemiological data became reliably available. Furthermore, these baseline levels of LLIN supply were routinely topped up through continuous distribution channels. They were also supplemented every few years with further mass distribution campaigns, so coverage most probably remained reasonably high throughout the period of observation. Therefore, the findings presented herein should not be interpreted as inferring any lack of impact of LLINs upon epidemiological outcomes [26,56]. Rather it likely reflects the already-high baseline coverage achieved through these initial campaigns before systematic incidence monitoring began using HMIS.

Indeed, it is noteworthy that a long-term decline in malaria cases began even before the introduction of IRS from 2010 through to 2014 (Fig 3), although this downward trend could not be clearly confirmed by interrupted time series analysis (Table 3). The earliest distributions of LLINs may therefore have contributed to this apparent consistent downward trend over the first few years of the study, which was most evident early on in catchments that did not receive IRS until 2014 (Fig 3). However, the impressive overall decline that occurred over the full duration of the study, is unlikely to have arisen solely from prior LLIN scale up, which is likely to have taken more than three years to achieve its full effect upon malaria incidence [42–45]. More effective testing and treatment, through improved access to RDTs and artemisinin-based combination therapies [14] respectively, may have also contributed to this encouraging long term trend and the long-term impact of simultaneously opening so many new health facilities upon that trajectory is very clear from mid-2016 onwards (Fig 3, Table 3).

Reassuringly, another study using routine HMIS data from all across Zambia indicated that LLINs did indeed achieve substantial impacts upon malaria incidence [39], although similar effects upon malaria-related mortality could only be clearly confirmed in urban districts [57]. Beyond Zambia, a plethora of other studies from all across sub-Saharan Africa, up to and including several cluster-randomized trials, have also confirmed that LLINs have demonstrable impacts upon epidemiological endpoints [26,58].

Having said that, of course, the limitations of LLINs are also well understood [59]. Limited physical durability, access, and utilisation [26], coupled with outdoor feeding and resting by vectors of residual malaria transmission like *An. arabiensis* [5], constrain reasonable expectations of this invaluable but imperfect vector control tool [60–62]. Also, the growing resistance to pyrethroid insecticides across Africa, which has been clearly documented in this particular study area [7], now clearly undermines the impacts of LLINs all across the continent [59,63–65]. As a result, it has become increasingly obvious over recent years that IRS with complementary active ingredients can help address this growing shortcoming of pyrethroid-only LLINs, even though both interventions target mosquitoes indoors [56]. The results presented herein clearly confirm such directly attributable immediate incremental benefits lasting up to a year after the supplementation of LLINs with IRS using non-pyrethroid insecticides (Tables 1 to 3 and Figs 2 and 3).

Although malaria incidence may have already been trending downward prior to IRS introduction, spraying clearly achieved immediate additional short term transmission suppression for up to a year, especially when durably alternatives to pyrethroids were used. Indeed, the differential effects observed across insecticide formulations also underscore the importance of product selection in maximizing the impact of IRS programs. For example, IRS with the long-lasting organophosphate product PM-CS apparently reduced both malaria inpatient admissions and outpatient cases for an entire year after application (Tables 1 and 2). Regarding inpatient admissions to hospital with severe malaria, IRS with DC was only clearly associated with reduced case numbers for the first three months after spraying (Table 1) but a similar effect upon outpatient malaria cases was clearly attributable to the same supplementary intervention for a full year (Table 2). Given that the only difference between these two GLMM analyses was the epidemiological indicator used as the dependent variable, the lack of apparent impact upon severe cases beyond 3 months post-spray is most likely a type II statistical error arising from the limited statistical power that can be leveraged from records of the former rarer and more extreme outcome of infection.

The three apparent resurgences of severe malaria that interrupted the otherwise consistent long-term decline of inpatient admissions to Luangwa District Hospital could only be descriptively associated with various gaps in coverage with LLINs or IRS. Nevertheless, such rebounds [66] have been documented elsewhere after IRS with non-pyrethroids was withdrawn, reverting to exclusive reliance upon pyrethroid-only LLINs as the sole vector control measure [67,68]. The evidence presented herein confirms that, wherever they can resist growing budgetary pressures to do so, malaria control programmes should try to avoid scaling back this particular combination of vector control measures. Indeed, in an era of universal resistance to pyrethroids, the assumption that high LLIN coverage renders supplementary IRS redundant is clearly outdated [26,66].

While the most obvious explanation for the surprising apparent association of IRS with DM-WG with increased inpatient admissions from 7 to 12 months after application (Table 1) is simply a type I error, such counterintuitive trends have been recorded by community-based mosquito and parasite survey platforms in previous studies of the same area [7]. Although this phenomenon might be alternatively explained by the deterrent properties of this insecticide to mosquitoes [69,70], it is difficult to envision how this would only become relevant more than 6 months after spraying. This IRS treatment appeared to reduce outpatient malaria cases by two fifths over the first 3 months after spraying, although that apparent effect only approached significance and no similar effect was apparent at any time thereafter (Table 2). This more common outcome of infection obviously affords modelling analyses greater statistical power, so the results based on total cases (Table 2) cast further doubt on the authenticity of the multivariate model fitted to the much smaller numbers of severe cases (Table 1). For the other pyrethroid-based LC-CS product, the only detectable effect was a reduction of outpatient cases by over half, which was surprisingly only observed for 7–12 months after spraying. Again, this unexpected timing of the inferred effect may also represent a spurious model fit rather than a genuine interventional effect.

Interestingly, the consistent epidemiological effects estimated for the long-lasting PM-CS formulation over the course of a full year, and those detected for DC in relation to outpatient cases are noteworthy. These findings suggest that that these formulations can extend control well beyond the duration of the rainy season and the transmission peak that immediately follows it. Such options for year-round transmission suppression may enhance the impacts of complementary drug-based approaches for clearing the infectious reservoir [71]. This may be especially important in areas where *An. funestus.s.s.,* can sustain intense transmission through the dry season [23,72].

In this setting, resistance to pyrethroids became apparent quite early in the course of this observation period [3,7], necessitating the subsequent switch from pyrethroids to other alternative insecticide classes to which vector populations still remain susceptible. The finding that supplementing LLINs with non-pyrethroid IRS further reduces malaria burden is therefore readily rationalized, not to mention consistent with several other studies across Africa [73–76]. While this augments the rationale for supplementing pyrethroid-based LLINs with non-pyrethroid IRS, these two interventions should nevertheless be combined judiciously within the constrained resource envelopes of national control programmes. Also, wherever budget limitations constrain coverage with the two, the highest priority should be given to areas with the highest levels of malaria transmission and pyrethroid resistance [26]. Careful consideration should also be given to only using insecticide combinations that are complementary to each other in the context of local resistance profiles, rather than redundant or even antagonistic [2,26,76].

Of course, like any epidemiological study, this one has substantial limitations, the most obvious of which is simply that it was undertaken on a retrospective and observational basis, rather than prospectively planned and experimentally controlled. Therefore, it only provides evidence for the plausibility of the insights obtained rather than their probability *per se* [77,78]. Also, this opportunistic use of routinely collected legacy data from the HMIS data has several limitations of its own, such as over-reporting and under-reporting of cases in a manner that likely varies from one facility to another. While the quality of this data was likely improved over time by regular training of health workers, upgrades of HMIS platforms and the introduction of robust internal validation measures, these changes may have contributed to systematic biases that could have somewhat distorted the longitudinal trends apparent in Figs 2 and 3 and Table 3.

Indeed, the large increase in both total and severe malaria incidence cases in 2010 and 2011, relative to 2009, is notable and may well be explained by the nationwide scale up of RDTs and a new HMIS platform at the time [14,39]. Furthermore, between November 2010 and February 2013, the high numbers of inpatient admissions could have also been stimulated by improved access to diagnosis and treatment services through trained community health workers (CHWs). This new cadre of community-based decentralized delivery of basic healthcare services was alsooperationalized in Luangwa through a pilot evaluation around seven of these nine health facilities. Indeed, similar increases of hospitalization rates in northern Zambia have been attributed to introduction of community case management through CHWs, which apparently led to increased inpatient admissions arising from enhanced referral rates from communities [79]. On the other hand, the rise of case numbers in 2010 might also be partly explained by the fact that vector control interventions were disrupted following delays in the disbursement of programmatic funds [15].

Another notable limitation of the study is the exclusion of eight health facilities that were established from mid-2016, to ensure consistent coverage of the full study period across all units of observation. However, the pooling of inpatient admission data from Luangwa Boma Rural Health Centre with that obtained from the new Luangwa District Hospital appears to cope with that sudden change without noticeably disrupting trends in recorded cases of severe malaria (Fig 2). Regarding total outpatient cases, the clearly attributable effect of simultaneously opening the 8 new facilities in mid-2016 (Fig 3, Table 3) is very encouraging regarding the long-term knock-on effects of scaling up diagnosis and treatment services upon malaria burden and transmission.

## Conclusions

Despite all the caveats discussed above, no compelling reasons to disregard the broad picture painted by these data are obvious. The statistical attribution of immediate incremental reductions to IRS with durable non-pyrethroid insecticide formulations and long-term benefits of scaling up access to clinical services reported herein, should therefore probably be interpreted at approximate face value.

While no effect of LLINs upon either inpatient or outpatient cases could be statistically attributed to fluctuating rates of intervention delivery, this should not be interpreted as inferring any lack of effectiveness of this essential life-saving intervention. This is because the district had already received substantial numbers of nets before reliable incidence monitoring began, and coverage was subsequently maintained by several large-scale distributions occurred over the course of the study. Together with improved diagnostic and treatment services, the scale up of LLINs before this study began may have contributed to the apparent downward trajectory in malaria burden over the first few years of the study (Fig 3), although this trend could not be confirmed by interrupted times series analysis of the full data set (Table 3).

What is clear, however, is that substantial incremental reductions of malaria incidence could be directly attributed to some of the IRS treatments for up to 12 months, especially long-lasting formulations of non-pyrethroid active ingredients. Even though malaria cases were already declining, the introduction of IRS clearly consolidated these gains further. Furthermore, the opening of an additional 8 new health facilities at the same time clearly triggered a further change in trajectory across the district, with malaria incidence steadily dissipating from that point forward. As a result, overall inpatient and outpatient incidence across the entire district slowly dropped to <10% and <20% respectively, relative to their historical peak levels reported soon after the start of the observation period.

## Abbreviations

ANC: antenatal clinic
CHW: community health workers
CS: capsule suspension
DM: deltamethrin
DC: deltamethrin-clothianidin co-formulation
EC: emulsifiable concentrate
EPI: expanded programme for immunization
GLMM: generalised linear mixed models
GPIRM: global plan for insecticide resistance management
HMIS: health management information system
IRS: indoor residual spraying
LC: lambda-cyhalothrin
MRRS: malaria rapid reporting system
LLIN: long lasting insecticidal treated nets
PBO: piperonyl butoxide
PM: pirimiphos-methyl
WG: wettable granule.
